# Sex-Dependent End-of-Life Mental and Vascular Scenarios for Compensatory Mechanisms in Mice with Normal and AD-Neurodegenerative Aging

**DOI:** 10.3390/biomedicines9020111

**Published:** 2021-01-24

**Authors:** Aida Muntsant, Francesc Jiménez-Altayó, Lidia Puertas-Umbert, Elena Jiménez-Xarrie, Elisabet Vila, Lydia Giménez-Llort

**Affiliations:** 1 Department of Psychiatry and Forensic Medicine, School of Medicine, Universitat Autònoma de Barcelona, 08193 Barcelona, Spain; aida.muntsant@uab.cat; 2 Institut de Neurociències, Universitat Autònoma de Barcelona, 08193 Barcelona, Spain; 3 Department of Pharmacology, Toxicology and Therapeutics, School of Medicine, Universitat Autònoma de Barcelona, 08193 Barcelona, Spain; lidiapu28@gmail.com (L.P.-U.); elisabet.vila@uab.cat (E.V.); 4 Stroke Unit, Department of Neurology, Institut d’Investigació Biomèdica (IIB)-Sant Pau, 08041 Barcelona, Spain; ejimenezx@santpau.cat

**Keywords:** healthy life expectancy (HALE), morbidity/mortality paradox, anxiety, cognition, systolic blood pressure, cerebral blood flow, arterial properties, angiogenesis, gender medicine, neurodegenerative disorders

## Abstract

Life expectancy decreases with aging, with cardiovascular, mental health, and neurodegenerative disorders strongly contributing to the total disability-adjusted life years. Interestingly, the morbidity/mortality paradox points to females having a worse healthy life expectancy. Since bidirectional interactions between cardiovascular and Alzheimer’s diseases (AD) have been reported, the study of this emerging field is promising. In the present work, we further explored the cardiovascular–brain interactions in mice survivors of two cohorts of non-transgenic and 3xTg-AD mice, including both sexes, to investigate the frailty/survival through their life span. Survival, monitored from birth, showed exceptionally worse mortality rates in females than males, independently of the genotype. This mortality selection provided a “survivors” cohort that could unveil brain–cardiovascular interaction mechanisms relevant for normal and neurodegenerative aging processes restricted to long-lived animals. The results show sex-dependent distinct physical (worse in 3xTg-AD males), neuropsychiatric-like and cognitive phenotypes (worse in 3xTg-AD females), and hypothalamic–pituitary–adrenal (HPA) axis activation (higher in females), with higher cerebral blood flow and improved cardiovascular phenotype in 3xTg-AD female mice survivors. The present study provides an experimental scenario to study the suggested potential compensatory hemodynamic mechanisms in end-of-life dementia, which is sex-dependent and can be a target for pharmacological and non-pharmacological interventions.

## 1. Introduction

In a life expectancy that decreases with the aging process, cardiovascular, mental health, and neurodegenerative disorders strongly contribute to the total disability-adjusted life years (DALYs) with significant sex differences observed [1,2]. On the other hand, the morbidity/mortality paradox points to females having greater longevity but worse healthy life expectancy (HALE) than males [3]. Neurodegenerative disorders such as dementia are associated with increased mortality compared to aged control populations [4,5,6]. Besides, in recent years, the high degree of heterogeneity in the clinical and temporal patterns of advanced stages of Alzheimer’s disease (AD) in the elderly population evidences the existence of several subgroups of patients and demands clinical prognosticators of end-of-life dementia [7]. At the translational level, the shorter life span of animal models provides a particular scenario for studying and long-term monitoring of the factors relevant for health/disease and those factors involved in its fine-tuning modulation, from genetic and epigenetic to morphological, structural, and functional levels.

Among the animal models of AD, we have proposed long-term survivors of the widely used 3xTg-AD mice as a model for mortality selection bias and heterogeneity in end-of-life dementia [8]. This model, homozygous for the familial AD mutations PS1/M146V and APPSwe, also harboring the tauP301L human transgene, progressively develops temporal- and regional-specific neuropathological patterns and other hallmarks of the human disease [9,10,11]. The mortality rates of 3xTg-AD mice at 15 months of age, an advanced stage of amyloid and tau neuropathology, are higher than in the non-transgenic (NTg) counterparts [12]. Thus, survivors could be used to investigate the frailty/survival paradigm in normal and pathological aging, since a small number of animals overcome advanced neuropathological stages of the disease. Across the literature, most experimental research of our and other laboratories has shown higher mortality rates in homozygous [12,13] and heterozygous [14] male 3xTg-AD mice, despite female 3xTg-AD mice exhibiting worse neuropathological status than males [15].

Peripheral cardiovascular dysfunction as a risk factor in AD is among the promising emerging fields, since bidirectional interactions have been reported in these patients. Thus, amyloid pathology affects patients’ hearts [16], and the impact of cerebrovascular dysfunction independently of cerebral amyloid angiopathy has also been demonstrated [17]. The arterial function is crucial to regulate blood pressure and flow through the body by the contraction and relaxation of the vascular smooth muscle cells. Increasing evidence obtained in different mouse models suggests that AD is associated with vascular dysfunction affecting different circulation arterial beds [18,19,20,21]. Angiogenesis, or growth of new blood vessels from pre-existent vessels, is a fundamental process, such as during development, wound healing, and restoring blood flow from hypoxic regions. Previous studies support a role of angiogenesis as a compensatory response to impaired cerebral blood flow (CBF) in AD [22]. However, extensive angiogenesis can lead to increased vascular permeability and subsequent hypervascularization and brain damage in AD [23,24]. Overall, although the “vascular hypothesis” of AD is increasingly being understood, the influence of sex on the vascular features present in AD has been a neglected topic.

Cardiovascular disease resulting from oxidative stress inflammation can exacerbate Alzheimer’s disease. We recently reported the first evidence of sex-dependent worse physiologically relevant structural (increased passive external and internal diameters, cross sectional area) and functional (increased active internal diameters) properties in small peripheral mesenteric resistance arteries (MRA) in 15-month-old 3xTg-AD mice (advanced stages of disease) compared to age-matched mice with normal aging [21]. Thus, at both physiological and high intraluminal pressures, vascular alterations of female 3xTg-AD mice were found more pronounced than those found in age-matched male 3xTg-AD mice. Besides, a correlation between MRA properties and anxiety-like behavioral profile was found in both 3xTg-AD mice and age-matched non-transgenic counterparts with normal aging, pointing at the relevant interaction between vascular and mental health in the aging process.

The present work aimed to further explore the cardiovascular–brain interactions in normal and AD-neurodegenerative aging models using a life span paradigm. For that purpose, we studied two cohorts of NTg and 3xTg-AD mice, including both sexes, until the end of life. Survival, monitored from birth, showed exceptional worse mortality rates in females than males, independently of the genotype. This mortality selection provided a “survivors” sample that could unveil brain–cardiovascular interaction mechanisms relevant for normal and neurodegenerative aging processes in long-lived animals. The results show sex-dependent distinct physical (worse in 3xTg-AD males), neuropsychiatric-like and cognitive phenotypes (worse in 3xTg-AD females), and hypothalamic–pituitary–adrenal (HPA) axis activation (higher in females), with higher cerebral blood flow and improved cardiovascular phenotype in 3xTg-AD female mice survivors. The present study suggests a potential compensatory hemodynamic mechanism in end-of-life dementia, which is sex-dependent and can be a target for pharmacological and non-pharmacological interventions.

## 2. Materials and Methods

### 2.1. Animals

Homozygous triple-transgenic 3xTg-AD mice harboring human PS1/_M146V_, APP_Swe_, and tau_P301L_ transgenes were genetically engineered at the University of California Irvine, as previously described [9]. Briefly, two independent transgenes (encoding human APP_Swe_ and human tauP301L, both under control of the mouse Thy1.2 regulatory element) were co-injected into single-cell embryos harvested from homozygous mutant PS1M146V knock-in (PS1KI) mice. The PS1 knock-in mice were originally generated after embryonic transfer into pure C57BL/6.

A total of thirty-six 14-month-old homozygous male and female mice from the Spanish colonies of 3xTg-AD (*n* = 19, 10 males, 9 females) and C57BL/6 (*n* = 17, 10 males and 7 females) wild-type mice (from now, referred as non-transgenic mice, NTg) from litters of a breeding program established after embryonic transfer to C57BL/6 strain background were used in this study. All the animals were housed three to four per cage and maintained (Makrolon, 35 × 35 × 25 cm ^3^) under standard laboratory conditions (12 h light/dark, cycle starting at 8:00 h, food and water available ad libitum, 22 ± 2 °C, 50–60% humidity) at the Universitat Autònoma de Barcelona. Behavioral tests were performed from 9:00 h to 13:00 h. Assessments were performed blind to the experiment in a counterbalanced manner.

All procedures were in accordance with Spanish legislation on “Protection of Animals Used for Experimental and Other Scientific Purposes” and the EU Council directive (2010/63/EU) on this subject. The protocol CEEAH 3588/DMAH 9452 was approved the 8th of March 2019 by Departament de Medi Ambient i Habitatge, Generalitat de Catalunya. The study complies with the ARRIVE guidelines developed by the NC3Rs and aims to reduce the number of animals used [25].

### 2.2. Experimental Design

A longitudinal study divided into successive phases was performed, starting at 14 months of age, with the characterization of their physical and mental health status (behavioral phenotype and physical condition (weight, Mouse Clinical Frailty Index Assessment, and Survival) 441.7 ± 1.55 days (14.52 months). Thereafter, different physiological variables were evaluated: systolic blood pressure 470.9 ± 1.41 days (15.48 months); relative cerebral blood flow 477.9 ± 1.49 days (15.71 months); aortic function and angiogenesis 499.6 ± 2.14 days (16.43 month). Survival was continuously monitored, and glucocorticoid levels, as an indicator of HPA axis function, were analyzed from blood samples collected during the euthanasia.

### 2.3. Behavioral

At 14 months of age, a comprehensive screening of physical, emotional, and cognitive functions was successively performed. A battery of 7 tests was used, based on three main behavioral dimensions [12] that can be described as follows:

#### 2.3.1. Neuropsychiatric-Like Behaviors

Changes in emotionality increased neophobia, and other signs of anxiety-like responses, all of them BPSD-like behaviors modeled in 3xTg-AD mice [10], were measured in classical unconditioned tests. The test evaluates locomotion/exploration, anxiety-like behaviors, and emotionality under three different anxiogenic conditions: mild neophobia in a new home-cage (corner test), direct exposure to an illuminated field (open-field test), a choice between a dark and a lit chamber (dark–light box test), black corridors of a maze resembling burrows (T-maze test), and interaction with small objects (marble burying test).

#### 2.3.2. Corner Test (CT) and Open-Field Test (OF)

Neophobia was assessed in the corner test for 30 s. Animals were individually placed in the center of a clean standard home cage, filled with wood save bedding. The number of corners visited were recorded during 30 s [26]. Latency to realize the first rearing and the number of rearings were also registered [10]. Immediately after the CT, mice were placed in the center of an open field (metalwork, white box, 42 × 38 × 15  cm^3^) and observed for 5 min [27]. The ethogram, described by the temporal profile of the following sequence of behavioral events, was recorded: duration of freezing behavior, latency to leave the central square, and that of entering the peripheral ring and latency and total duration of self-grooming behavior. Horizontal (crossings of 10 × 10 cm^2^ squares) and vertical (rearings with wall support) locomotor activities were also measured. During the tests, defecation boli and urination were also recorded. The repeated test, 24 h later, was used to evaluate the long-term memory of these experiences [28].

#### 2.3.3. Dark–Light Box Test (DLB)

Anxiety and risk assessment were measured for 5 min after introducing the animals into the dark compartment of the DLB (Panlab, S.L., Barcelona, Spain). The apparatus consisted of two compartments (black, 27 × 18 × 27 cm^3^, white, 27 × 27 × 27 cm^3^ illuminated by a red 20 W bulb) connected by an opening (7 × 7 cm^3^). The experimental room was kept in darkness (without illumination). Latency to enter, time spent, and the number of entries into the lit compartment were recorded. The number of stretch attendances and self-grooming were also recorded.

#### 2.3.4. Marble Burying Test (MB)

Mice were placed individually in a standard home cage containing nine glass marbles (dimensions 1 × 1 × 1 cm^3^) evenly spaced making a square (three rows of three marbles per row only in the left area of the cage) on a 5 cm thick layer of sawdust. The mice were left in the cage with marbles for a 30 min period after which the test was terminated by removing the mice and counting the number of marbles: intact (untouched), rotated (90 or 180°), half-buried (at least ½ buried by sawdust), and buried (completely hidden).

#### 2.3.5. T-maze Test (TM)

Two different paradigms were carried out in a T-shaped maze (woodwork; two short arms of 30 × 10 cm^2^ and a long arm of 50 × 10 cm^2^). Copying with stress strategies, risk assessment, and working memory were assessed in a spontaneous alternation task [29]. Animals were placed inside the maze’s long arm with its head facing the end wall, and it was allowed to explore the maze during a maximum of 5 min. The latencies to each one of the goals in this task, namely, to move and turn (freezing behavior), then to reach the intersection, the time elapsed until the animal crossed (4 paws criteria) the intersection of the three arms, and the total time invested in exploring the three arms of the maze (test completion criteria) were recorded. The entry of an already visited arm in the trial before completing the test was considered an error. Defecation boli and urination were also noted.

The working memory paradigm was studied 24 h later and consisted of two consecutive trials: one forced choice followed, 60 s later, by one free choice (recall trial). In this case, mice were placed inside the short arm of the maze and the latencies to each one of the goals in this task, namely, to move and turn, then to reach the intersection, the time elapsed until the animal crossed (4 paws criteria) the intersection of the three arms and the time elapsed until the mice completed 20 s in the forced arm were recorded (time to reach the criteria). Sixty seconds later, the animals that completed the forced trial in less than the cut-off time (10 min) were allowed to explore the maze in a free choice trial where both arms were accessible for 5 min. The arm chosen by the mice and the time spent to reach the correct arm during the free choice were recorded (exploration criteria). The choice of the already visited arm in the previous trial was considered as an error, and the total number was calculated. Finally, defecation boli and urination were also recorded.

#### 2.3.6. Morris Water Maze Test (MWM)

Animals were tested for spatial learning and memory in the MWM test consisting of 1 day of cue learning and 2 days of place learning for spatial reference memory. We used this short protocol, adapted from the 2 day water maze protocol [30], as more suitable in studies where the repeated swimming or the water maze situation—which is stressful for mice but not for rats—can have an impact on other variables, such as cardiovascular system or blood pressure. Mice were trained to locate a hidden platform (7 cm diameter, 1 cm below the water surface) in a circular pool for mice (120 cm in diameter and 60 cm deep, 25 °C opaque water). Mice that failed to find the platform within 60 s were placed on it for 10 s, the same period as was allowed for the successful animals.

Cue learning with a visible platform: On the first day, the animals were tested for the cue learning of a visual platform consisting of four trials in 1 day. In each trial, the mouse was gently released (facing the wall) from one randomly selected starting point (W-S-E-N) and allowed to swim until it escaped onto the platform, elevated 1 cm above the water level in the NE position and indicated by a visible striped flag (5.3 × 8.3 × 15 cm^3^). Extra maze cues were absent in the black walls of the room.

Place learning with a hidden platform: On the following day, the place learning task consisted of four trial sessions per day for 2 days with trials spaced 30 min apart. The mouse was gently released (facing the wall) from one randomly selected starting point (N-E-W-S; E-N-S-W) and allowed to swim until escaped onto the hidden platform, which was now located in the middle of the SW quadrant (reversal). Different geometric figures hung on each wall of the room were used as external visual clues.

Variables of time (escape latency), distance covered, and swimming speed were analyzed in all the tasks’ trials. The escape latency was readily measured with a stopwatch by an observer unaware of the animal’s genotype and confirmed during the subsequent video-tracking analysis (ANY-Maze v. 5.14, Stoelting, Dublin, Ireland).

### 2.4. Body Weight, Mouse Clinical Frailty Index Assessment, and Survival

After the behavioral assessment, the body weight was recorded. Frailty was assessed using an adaptation of the MCFI [31], including 30 “clinically” assessed non-invasive items. For 29 of these items, mice were given a score 0 if not presented, 0.5 if there was a mild deficit, and 1 for severe deficit. Weight was scored based on the number of standard deviations from a reference mean. The clinical evaluation included the integument, the physical/musculoskeletal system, the vestibulocochlear/auditory systems, the ocular and nasal systems, the digestive system, the urogenital system, the respiratory system, signs of discomfort, and body weight. Survival was recorded continuously with a daily cadence.

### 2.5. Systolic Blood Pressure

Measurement of systolic blood pressure was performed in conscious NTg and 3xTg-AD mice using the tail-cuff method (NIPREM 645; Cibertec, Madrid, Spain). The average systolic blood pressure of each mouse was determined from six consecutive measurements after habituation, as described [32].

### 2.6. MRI-ASL—Relative Cerebral Blood Flow

MRI was carried out at the joint nuclear magnetic resonance facility of the Universitat Autònoma de Barcelona and Centro de Investigación Biomédica en Red—Bioingeniería, Biomateriales y Nanomedicina (CIBER-BBN) (Cerdanyola del Vallès, Spain) in a 7-Tesla horizontal magnet (BioSpec 70/30, Bruker BioSpin, Ettlingen Germany), equipped with actively shielded gradients (B-GA12 gradient coil inserted into a B-GA20S gradient system). For signal reception, a mouse brain surface coil was used actively decoupled from a 72 mm inner diameter volume resonator. Animals were anaesthetized using an average 2.5% isoflurane in O2, and both animal respiration and temperature were constantly monitored with a preclinical monitoring and gating system (SA Instruments, New York, USA). High-resolution T2-weighted images (T2w) were acquired for anatomical references using rapid acquisition with relaxation enhancement sequence with double echoes. The acquisition parameters were the following: orientation = axial plane, echo train length or rare factor = 8, field of view (FOV) = 1.92 × 1.92 cm^2^, matrix size (MTX) = 256 × 256 (75 × 75 μm/pixel), number of slices = 11, slice thickness = 1 mm, interslice distance = 1 mm, repetition time (TR)/ effective echo time (TEeff) = 5000/36 ms, number of averages = 1, and total acquisition time (TAT) = 2 min. Perfusion-weighted imaging was obtained using the MRI arterial spin labelling (MRI-ASL) technique without contrast. Two consecutive axial slices were placed in two different sections (Bregma −1.5 mm, −2.5 mm) according to the mouse brain atlas by Paxinos and Franklin [33] using a T2w sagital plane image as anatomic reference. The vendor provided ASL protocol using a flow-sensitive alternating inversion-recovery rapid acquisition with relaxation enhancement (FAIR-RARE) sequence. The parameters were the following: echo train length = 72, TR/TEeff = 16,000/ 50 ms, slice thickness = 1 mm, thickness of the selective inversion slice = 4 mm, FOV = 1.92 × 1.92 cm^2^ (150 × 150 μm/ pixel), MTX = 128 × 128, inversion recovery time (TIR) = 30 ms, increment of TIR = 100 ms, number of TIR = 22, and TAT = 13 min. The obtained ASL images were analyzed using the workstation software Paravision 5.1 (Bruker Española S.A., Madrid, Spain) to generate rCBF images. rCBF values were measured using a region of interest (ROI) created corresponding to cortex (Bregma −1.5 mm, −2.5 mm), striatum (Bregma −1.5 mm, −2.5 mm), caudate putamen (Bregma −1.5 mm), basolateral amygdala (-1.5 mm), and hippocampus (−2.5 mm) in both hemispheres (Figure 5). Bilateral ROIs from the same mouse were analyzed together as a mean value and separately to evaluated left-right rCBF asymmetries between hemispheres. For correlations, asymmetry index (AI) defined as (right-left)/(right + left) * 100 was used.

### 2.7. Angiogenesis

Segments of MCA and the descending thoracic aorta were dissected in ice-cold physiological salt solution (PSS; composition in mM: NaCl 112.0; KCl 4.7; CaCl_2_ 2.5; KH_2_PO_4_ 1.1; MgSO_4_ 1.2; NaHCO_3_ 25.0 and glucose 11.1) supplemented with amphotericin B (15 mg/l) (Biowhittaker ^®^, Lonza, Basel, Switzer- land) and gentamicine (30mg/l) (Genta-gobens ^®^, Laboratorios Normon SA, Tres Cantos, Madrid, Spain) and gassed with 95% O_2_ and 5% CO_2_. Afterwards, vessels were immersed into Matrigel^®^ (50 μL; BD Bioscience, San Jose, CA, USA) following the protocol previously described [34,35]. Angiogenic growth was measured using an inverted microscope equipped with a camera (10× objective; TE2000 Nikon Eclipse-S, Nikon España, Madrid, Spain) at day 4, 5, 6, and 6 after seeding. The longest vessel sprouting from the artery’s outer surfacy (starting point) determined the angiogenic growth [34,35], and at least three fields per arterial segment were measured.

### 2.8. Aortic Function

Segments (2 mm) of the descending thoracic aorta were dissected free of fat and connective tissue in ice-cold PSS gassed with 95% O_2_ and 5% CO_2_ and set up on an isometric wire myograph (model 410 A; Danish Myo Technology, Aarhus, Denmark) filled with PSS (37 °C; 95% O_2_ and 5% CO_2_), as described [36]. The vessels were stretched to 6 mN and allowed to equilibrate for 45 min. Afterwards, aortas were contracted twice with 100 mM KCl, and after washing, vessels were left to equilibrate (30  min) before starting the experiments. Endothelial-dependent vasodilatations to acetylcholine (ACh; 10^−9^ to 10^−4^ M) and endothelial-independent vasodilatations to sodium nitroprusside (10^−10^ to 10^−3^ M) were performed in phenylephrine (Phe)-precontracted (70–100% of 100  mM KCl contraction) vessels. Contractile responses to the α_1_-adrenoceptors agonist Phe (10^−9^ to 3  ×  10^−4^ M) were also studied.

### 2.9. HPA Axis Endocrine Status

Blood samples were collected; plasma was obtained by centrifugation and stored at −80 °C until corticosterone analysis. Corticosterone content (ng/mL) was analyzed using a commercial kit (Corticosterone EIA Immunodiagnostic Systems Ltd., Boldon, UK) and read at 450 nm of absorbance with Varioskan LUX ESW 1.00.38 (Thermo Fisher Scientific, Massachusetts, MA, USA)

### 2.10. Statistics

Results are expressed as mean ± SEM. SPSS 15.0 (SPSS Inc., Chicago, IL, USA) and GraphPad Prism 5.0 (GraphPad Software Inc., San Diego, CA, USA) software were used. A 2 × 2 factorial design with multivariate general lineal model analysis evaluated genotype (G) and sex (S) effects. Two independent groups were compared with Student’s *t*-test, while comparisons for related samples were made with the paired *t*-test. The survival curve was analyzed with the Kaplan–Meier test. Relaxations to ACh and sodium nitroprusside were expressed as the percentage change from the Phe precontracted level. Contractions to Phe are expressed as a percentage of the tone generated by 100 mM KCl. The area under the curve was individually calculated from each concentration–response curve to ACh, sodium nitroprusside, and Phe and was expressed as arbitrary units. Differences between concentration–response curves were assessed by two-way repeated measures ANOVA with Tukey’s post-test. The correlations between the different variables studied were evaluated with Pearson’s correlation. In all the tests, *p* < 0.05 was considered statistically significant.

## 3. Results

### 3.1. Survival

**Figure 1:**
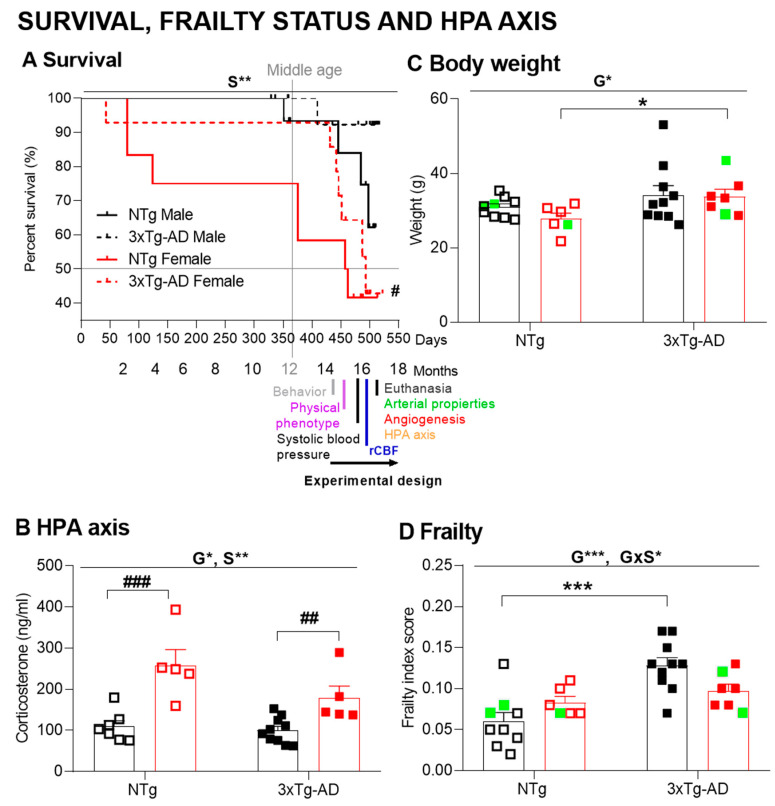
Survival, hypothalamic–pituitary–adrenal (HPA) axis endocrine status and physical health. (**A**) Survival; (**B**) corticosterone levels; physical health: (**C**) body weight, and (**D**) frailty scores of 14-month-old mice. Results are expressed as the mean ± SEM. Initial sample size: NTg, male *n* = 10, female *n* = 7; 3xTg-AD, male *n* = 10, female *n* = 9. Bars illustrate the genotype groups, as indicated in the abscissae. Symbols are used to illustrate individual values of males (black, left panel) and females (red, right panel). In green: the no-survivors. Statistics: 2 × 2 factorial ANOVA analysis design: genotype (G), sex (S) and genotype × sex (G×S) interaction effects, * *p* < 0.05, ** *p* < 0.01, *** *p* < 0.001 (above line). Student *t*-test comparisons: * *p* < 0.05, ** *p* < 0.01, *** *p* < 0.001 vs. the corresponding NTg group; ## *p* < 0.01, ### *p* < 0.001 vs. the corresponding male group.

Survival, from birth to 16 months of age, of an initial sample of fifty-nine mice (29 NTg, 17 males, 12 females; 30 3xTg-AD, 16 males; 14 females) is illustrated in Figure 1A. Log-rank analyses show an overall difference in survival curves over the four groups (χ2(3) = 10.634, *p* = 0.014). There was no significant difference between the curves for NTg male and female mice, but female 3xTg-AD mice had a shorter lifespan than male 3xTg-AD mice (χ2(1) = 5.168, *p* = 0.023). Pairwise comparisons in the survival curve confirmed the effect of sex factor with females’ worse survival, independently of the genotype (χ2(1) = 8.224, *p* = 0.004). Both groups of females exhibited an early 10% mortality rate before young adulthood (2 months of age), and their mean life expectancy was achieved at late adulthood (459 days or 15.1 months in NTg females, 493 days or 16.2 months in 3xTg-AD females). However, they differed in the temporal course of mortality, with a young adulthood and late adulthood mortality pattern in NTg and 3xTg-AD mice, respectively. In this cohort, the drop of survival for male NTg mice was at middle age (12 months), and at the end of the experiment (16 months), their survival rate was 62%. In contrast, the survival of male 3xTg-AD mice was 92%. Rates of censored data for each group ranged from 93.8 to 50%. Thirty-six animals started the experimental design, 17 NTg (10 males, seven females) mice, and 19 3xTg-AD (10 males, nine females). During the experimental research, three NTg males, two NTg females, and four 3xTg-AD females died.

### 3.2. HPA Axis Endocrine Status

A sex effect was observed with increased levels of corticosterone in the plasma of females (Sex (S), Factorial analysis (F) (1,27) = 30.015, *p <* 0.001) as compared to respective male counterparts (post hoc test, *p <* 0.006). This effect was more notorious in the NTg groups, leading to an overall genotype difference as well (Figure 1B) (G, F (1,27) = 4.634, *p* = 0.042).

### 3.3. Behavioral Assessment for Physical, Emotional, and Cognitive Phenotypes

At 14 months of age, a comprehensive screening of three main behavioral dimensions and functions, namely, physical, emotional, and cognitive, was performed using a battery of seven tests as previously described [37].

#### 3.3.1. Physical Phenotype

Genotype differences were found in body weight (Figure 1C), which was increased in 3xTg-AD mice (Genotype (G), F(1,32) = 5.204; *p* = 0.030), an effect that was more clearly observed among females (post hoc test, *p* < 0.05).

Frailty score (Figure 1D) was increased in 3xTg-AD mice (G, F(1,32)= 12.052, *p* = 0.002) with a statistically significant two-fold increase in male mice as compared to NTg counterparts (post hoc test, *p* = 0.001), while NTg and 3xTg-AD females exhibited similar frailty scores (GxS; F (1,32) = 6.136; *p*= 0.020).

#### 3.3.2. Neuropsychiatric symptoms (NPS)-like phenotype and cognitive impairment under different anxiogenic conditions

**Figure 2:**
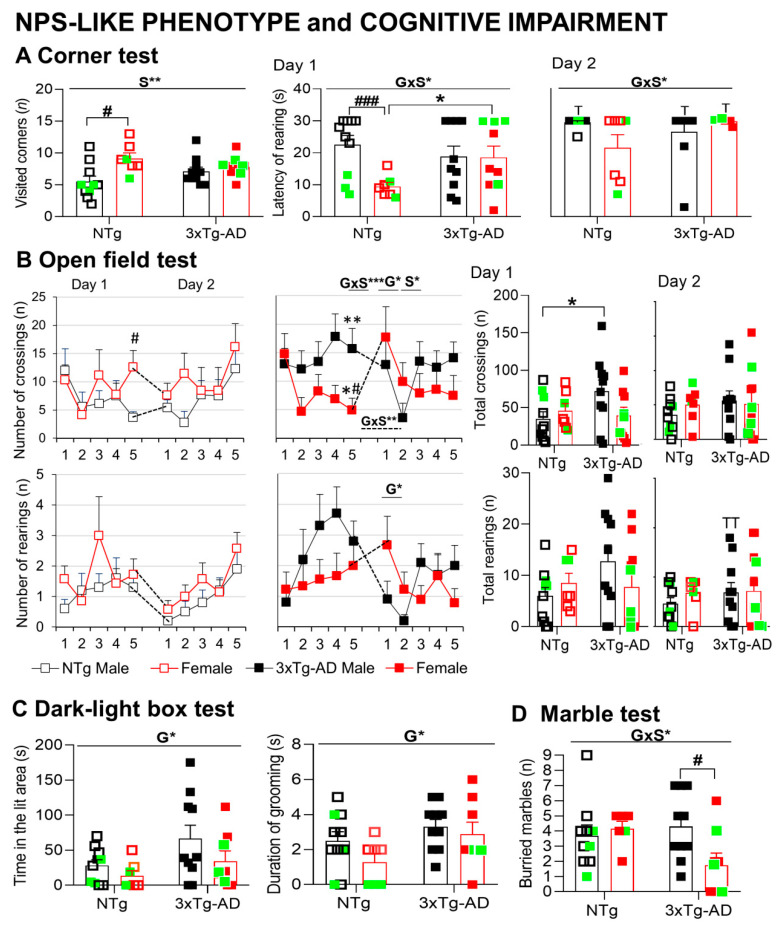
Mental health: Neuropsychiatric-like phenotype and cognitive impairment under different anxiogenic conditions. (**A**) Two-day corner test, (**B**) two-day open-field test, (**C**) dark–light box, (**D**) marble test. Results are the mean ± SEM. Bars illustrate the genotype groups, as indicated in the abscissae. Symbols are used to illustrate individual values of males (black, left panel) and females (red, right panel). In green: the no-survivors. Genotype (G) and sex (S) effects and GxS interaction were analyzed by 2 × 2 factorial ANOVA analysis, * *p* < 0.05, ** *p* < 0.01, *** *p* < 0.001 (above line). Time (T) factor (day-by-day) was analyzed by repeated measures ANOVA, ^T^ *p* < 0.05, ^TT^ *p* < 0.01, vs. the corresponding day 1 results. Student *t*-test comparisons: * *p* < 0.05, ** *p* < 0.01, *** *p* < 0.001 vs. the corresponding NTg group; # *p* < 0.05, ### *p* < 0.001 vs. the corresponding male group.

In the corner test (CT) for neophobia (Figure 2A), NTg females exhibited increased behavior, as measured by a higher number of visited corners and faster onset of rearing than NTg males (Student *t*-test, *p* < 0.05). In the 3xTg-AD mice, the behavior was slightly increased compared to male NTg response but did not reach statistical significance. Overall, the sex difference was shown in the visited corners (S, F(1,36) = 8.032, *p* = 0.008). Genotype per sex interaction effects in the variables for vertical exploratory behavior indicated the consistent results between male and female 3xTg-AD mice in this regard, while in the NTg mice, sex differences were shown (GxS, F(1,36) = 4.267, *p* = 0.047).

In the open-field (OF) test (Figure 2B), male and female NTg mice behaved quite similarly, as shown by the time course and total counts of their horizontal and vertical activities. In contrast, male 3xTg-AD mice exhibited sustained horizontal activity during the test and higher total counts than their NTg counterparts (OF1, repeated measures ANOVA (RMA); crossings: time × genotype × sex, F(1,36) < 0.001, *p* = 0.003). Female 3xTg-AD behaved like NTg mice, with a drop of activity from the first to the second minute of the test. These patterns resulted in statistically significant genotype x sex interaction effects (OF1 min 5, F(1,36) = 17.187, *p* = 0.001). When central and peripheral activity were distinguished, male 3xTg-AD mice showed an increased number of peripheral crossings in the third, fourth, and fifth minutes of the test (not shown) and, as a result, also on the total number, as compared to NTg animals and 3xTg-AD females.

In the repeated corner test (24 h later), all the groups showed lower levels of activity (paired *t*-test, *p* < 0.05). The genotype × sex factors interaction effects shown on the first day were also found here.

In the repeated open-field test (24 h later), the time course of horizontal and vertical activity was also dependent on the genotype and sex or both (OF2, RMA crossings: TxG, TxS and RMA rearing: TxG, TxS, TxGxS, all Fs (1,36) > 4.251.000, *p* < 0.047). NTg mice performed similar total activity levels than the precedent day. During the first minute of the repeated open-field test, performance did not differ from that shown in their first experience in the test (OF21 vs. OF11, *n.s*. in all the groups). Here, genotype effects on the number of crossings and rearing in the first minute of the test (F(1,36) > 4.584, *p* < 0.05) indicated higher performances in 3xTg-AD mice than NTg mice. Still, male 3xTg-AD reduced their total activity to control levels. 

A GxS effect was observed when we calculated the difference between the crossings performed in the first minute of the test on day2 (OF21) and those in the last minute of day 1 (OF15) (F(1,36) = 10.889, *p* = 0.002), with only female 3xTg-AD mice differing from their NTg counterparts (*p* < 0.05).

In the dark–light box (DLB) test (Figure 2C), 3xTg-AD mice exhibited a disinhibitory behavior, as shown by the increased number of crossings in the lit area (data not shown, G, F(1,35)= 5.186, *p* = 0.03) and increased total time spent into it (G, F(1,35) = 4.387, *p* = 0.044), as compared to the NTg genotype. Emotionality in 3xTg-AD mice was also increased, as they spent more time grooming (G, F(1,35) > 4.919, *p =* 0.034).

In the marble (MB) test (Figure 2D), 44.4% (4/9) of the marbles were buried on average by the groups except in the group of 3xTg-AD females that buried only 22.2% (2/9), resulting in a sex difference not found in NTg genotype (GxS, F (1,34) = 4.254, *p =* 0.048). A sex effect was also observed when we measured the marbles left intact (not shown, S, F (1,34) = 4.501, *p* = 0.042).

**Figure 3:**
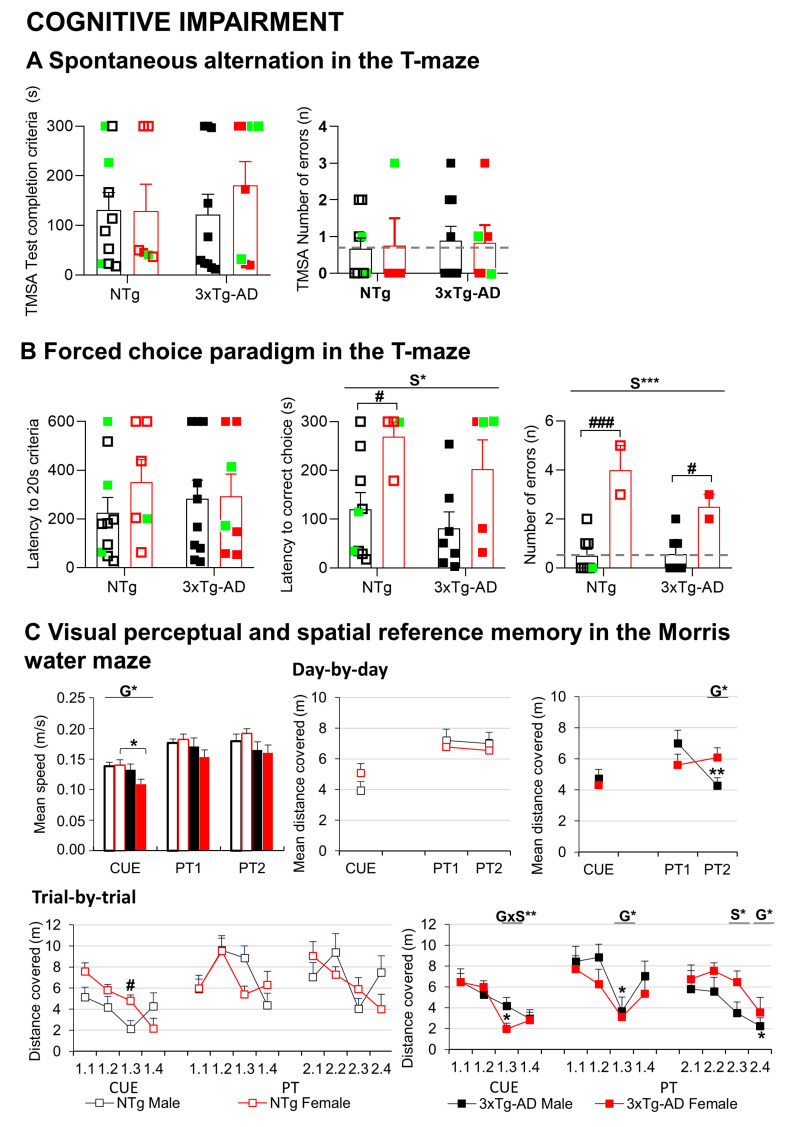
Mental health: cognitive impairment (**A**) Spontaneous alternation T-maze, (**B**) T-maze, (**C**) Morris water maze. Results are the mean ± SEM. Bars illustrate the genotype groups, as indicated in the abscissae. Symbols are used to illustrate individual values of males (black, left panel) and females (red, right panel). In green: the no-survivors. Genotype (G) and sex (S) effects and GxS interaction were analyzed by 2 × 2 factorial ANOVA analysis, * *p* < 0.05, ** *p* < 0.01, *** *p* < 0.001 (above line). Student *t*-test comparisons: * *p* < 0.05, ** *p* < 0.01, *** *p* < 0.001 vs. the corresponding NTg group; # *p* < 0.05, ### *p* < 0.001 vs. the corresponding male group.

Two different paradigms were carried out in a T-shaped maze, as depicted in Figure 3A; all the groups included animals that failed to complete the T-maze spontaneous alternation test (latency, 300 s) and the forced memory test (latency, 600 s). On average, all the groups showed similar latencies in the ethogram of behaviors exhibited in the T-maze spontaneous alternation (TMSA) test (turning, reaching the intersection, crossing the intersection with four paw criteria, completing the test) (G, S, all Fs (1,34) < 0.231; *p* > 0.05). The most representative of these latencies—that of achieving “test completion criteria”—is illustrated. The number of spatial working memory errors (revisiting an explored area) were recorded in those animals able to initiate the task. No statistically significant differences were observed between groups in spatial alternation.

In the second paradigm for working memory in the T-maze (TM) test (Figure 3B), all the groups needed the same time to reach the acquisition criteria in the forced trial (G, S, all Fs(1,33) < 0.708; *p* > 0.05). In the recall trial, considering the animals that completed the test (n = 25; nine NTg males; four NTg females; seven 3xTg-AD males and five 3xTg-AD females), sex differences, clearer among NTg, were found (S, F (1,25) = 10.063, *p* = 0.005), with males investing shorter times than females to reach the exploration criteria. Despite the small number of animals, an increased number of errors in working memory (revisiting an explored area) was noted in females (S, F (1,19) = 34.135, *p* < 0.001).

In the Morris water maze (MWM) (Figure 3C), genotype differences were found in the navigation speed, reaching statistically significant differences in the cue learning paradigm. Thus, 3xTg-AD mice were swimming slower than NTg counterparts (CUE: G, F (1,32) = 4.437, *p* = 0.044), an effect that was more clearly shown in females (*p* < 0.05). Therefore, the distance covered to reach the platform was used to illustrate all the paradigms’ performances. Genotype x sex interaction effects in the cue and the place tasks (PT) indicate that males’ and females’ performances were dependent on the genotype. Here, sex differences in the cue learning were shown in NTg mice, but not 3xTg-AD mice. Female NTg covered more distance than NTg males, whereas both sexes performed equally in the 3xTg-AD genotype. At the end of the four trial sessions of the cue learning task, all the groups could reach the platform in 20 s and cover 2 to 3 m.

In the two daily sessions of the place learning task, where the platform was hidden and located in a reversed position, animals exhibited a genotype effect in the distance covered to find the new location of the platform (PT11: G, F(1,32) = 6.228, *p* = 0.019). NTg mice were faster (shorter latency, not shown) and covered less distance to find the hidden platform. After that, the performances between NTg and 3xTg-AD mice differed in some trials. Genotype effect was also observed in the last trial of the second day; in this case, as in the mean distance of place task, two 3xTg-AD male mice performed less distance to arrive at the platform (PT24: G, F (1,32) = 4.964, *p* = 0.034; meanPT2, G, F(1,32) = 5.926, *p* = 0.022). The sex effect was observed in PT23, with females covering more distance to find the platform (PT23: S, F(1,32) = 4.716, *p* = 0.039).

In summary, different survival and behavioral signatures were found in these cohorts. Namely, (1) physical: An early mortality window of the female sex found enhanced in the AD-genotype and increased frailty only in male 3xTg-AD mice. (2) Neuropsychiatric-like: increased and persistent neophobia in female 3xTg-AD mice, a hyperactive pattern of male 3xTg-AD mice, and disinhibitory behavior in male and female 3xTg-AD mice. (3) Worse long-term memory of female 3xTg-AD in the open-field test; overall bad performances of all the animals in the mazes, with worse performance and working memory of female 3xTg-AD mice, a slower swimming speed of female 3xTg-AD mice, and paradoxical performances of male 3xg-AD mice, probably related to emotional and physical comorbidities. As discussed in previous work, these sex-dependent effects point at the relevance of the sex-specific analysis of AD disease. The results also illustrate the relevance of controlling for frailty and mortality rates to discriminate against the confounding factors (synopsis; Table 1).

### 3.4. Systolic Blood Pressure

**Figure 4:**
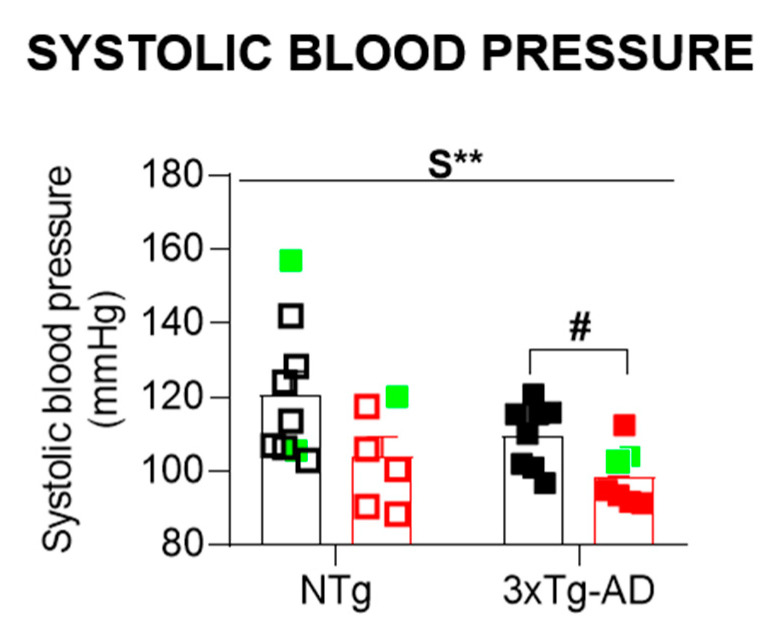
Systolic blood pressure. Results are the mean ± SEM. Bars illustrate the genotype groups, as indicated in the abscissae. Symbols are used to illustrate individual values of males (black, left panel) and females (red, right panel). In green: the no-survivors. Genotype (G) and sex (S) effects and GxS interaction were analyzed by 2 × 2 factorial ANOVA analysis, ** *p* < 0.01 (above line). Student *t*-test comparisons: ** *p* < 0.01, vs. the corresponding NTg group; # *p* < 0.05 vs. the corresponding male group.

Figure 4 illustrates the systolic blood pressure. Two transgenic animals could not be assessed because of a lack of vasodilation (1 animal) and overweight (1 animal). The sex effect was observed, with males presenting an increased systolic blood pressure compared to females (S, F (1,30) = 8.163, *p* = 0.008). These differences were especially observed between 3xTg-AD mice (Student *t-*test, *p* = 0.022).

### 3.5. MRI Relative Cerebral Blood Flow

**Figure 5:**
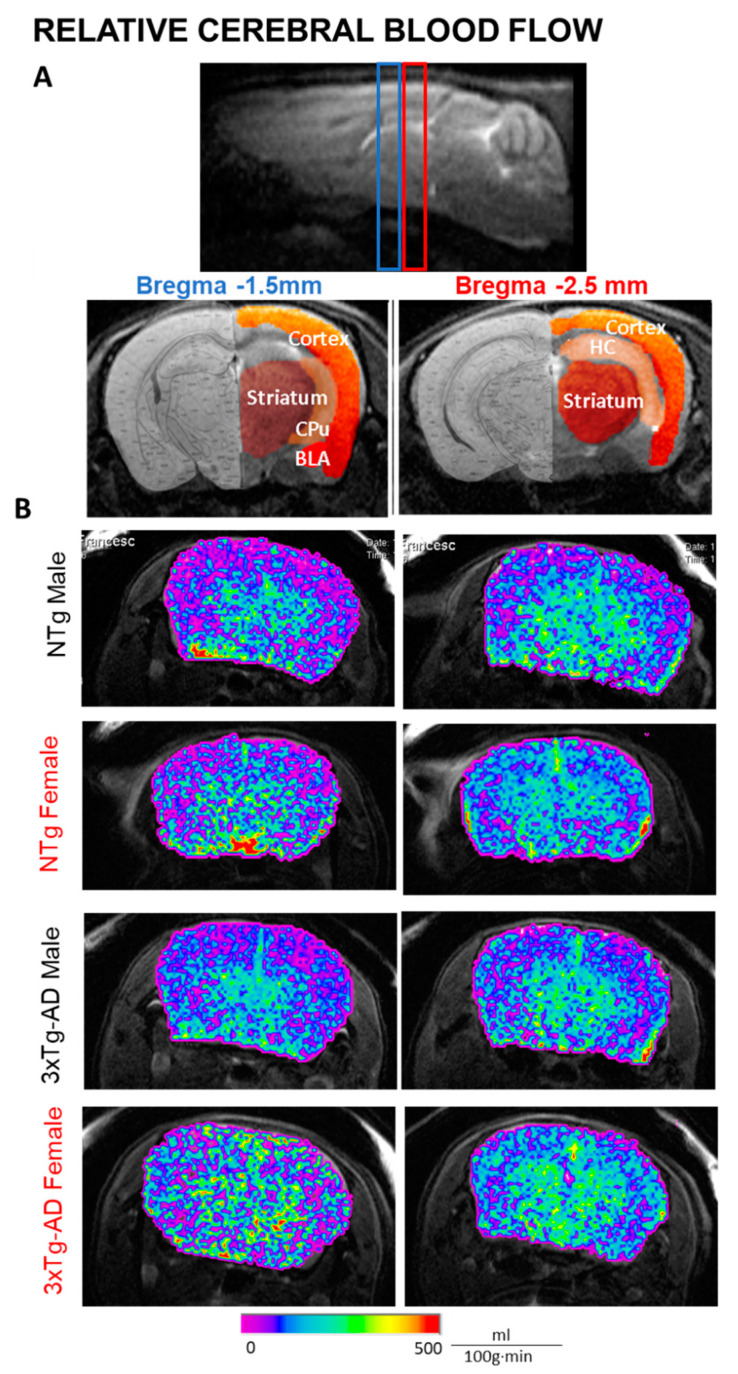
MRI arterial spin labeling (ASL)—relative cerebral blood flow in regions of interest. (**A**) Global cerebral blood flow and five regions of interest were examined in the present study from two different sections (Bregma −1.5 mm, −2.5 mm). Cortex; striatum; caudate putamen (CPu); basolateral amygdala (BLA); hippocampus (HC). (**B**) Representative images from rCBF maps superimposed to T2w-image from NTg male, NTg female, 3xTg-AD male, and 3xTg-AD female.

**Figure 6:**
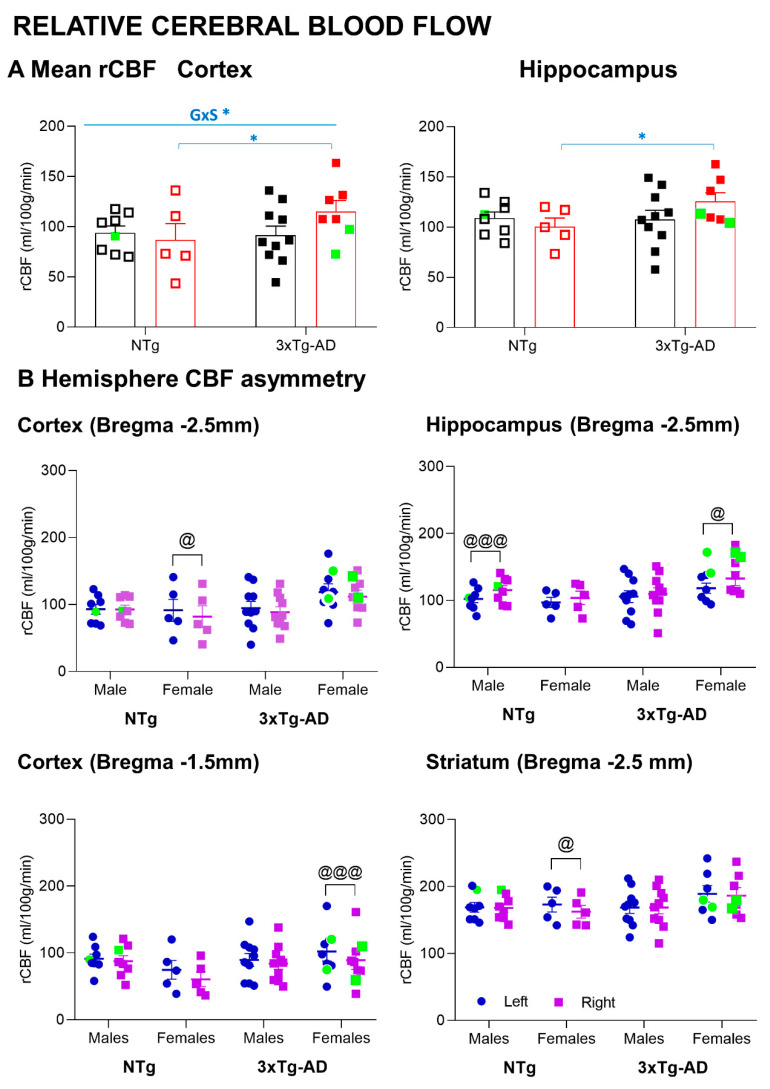
MRI-ASL—relative cerebral blood flow. (**A**) Mean relative blood flow in cortex and hippocampus. (**B**) Hemisphere relative cerebral blood flow asymmetry. Results are the mean ± SEM. Bars illustrate the genotype groups, as indicated in the abscissae. Symbols are used to illustrate individual values of males (black, left panel) and females (red, right panel). Left hemisphere: blue; right hemisphere: purple. In green: the no-survivors. Genotype (G) and sex (S) effects and GxS interaction were analyzed by 2x2 factorial ANOVA analysis, * *p* < 0.05 (above line). Student *t*-test comparisons: * *p* < 0.05 vs. the corresponding NTg group; Paired *t*-test in asymmetry between right/left hemispheres: @ *p* <0.05, @@@ *p* < 0.001.

Representative relative cerebral blood flow (rCBF) images in five regions of interest: cortex, striatum, hippocampus (HC), caudate-putamen (CPu), and basolateral amygdala (BLA) from two 1mm consecutive slices (approximately, Bregma −1.5 mm and −2.5mm) are presented in Figure 5. No statistically significant differences in rCBF were observed (Figure 6A). However, 3xTg-AD female mice survivors had increased rCBF in the cortex and hippocampus compared with their NTg counterparts (Student t-test *p* < 0.05). In particular, genotype per sex interaction effects were observed in cortical rCBF (GxS, F(1,27) = 4.545, *p* = 0.044).

For each group, the asymmetries in the rCBF of the left and right hemispheres are illustrated in Figure 6 B. In the cortex, rCBF asymmetries were found in females. The asymmetry between left–right hemispheres was also observed in the hippocampus of NTg males and 3xTg females and the striatum of NTg females (paired *t*-test, *p* < 0.05).

### 3.6. Angiogenesis

**Figure 7:**
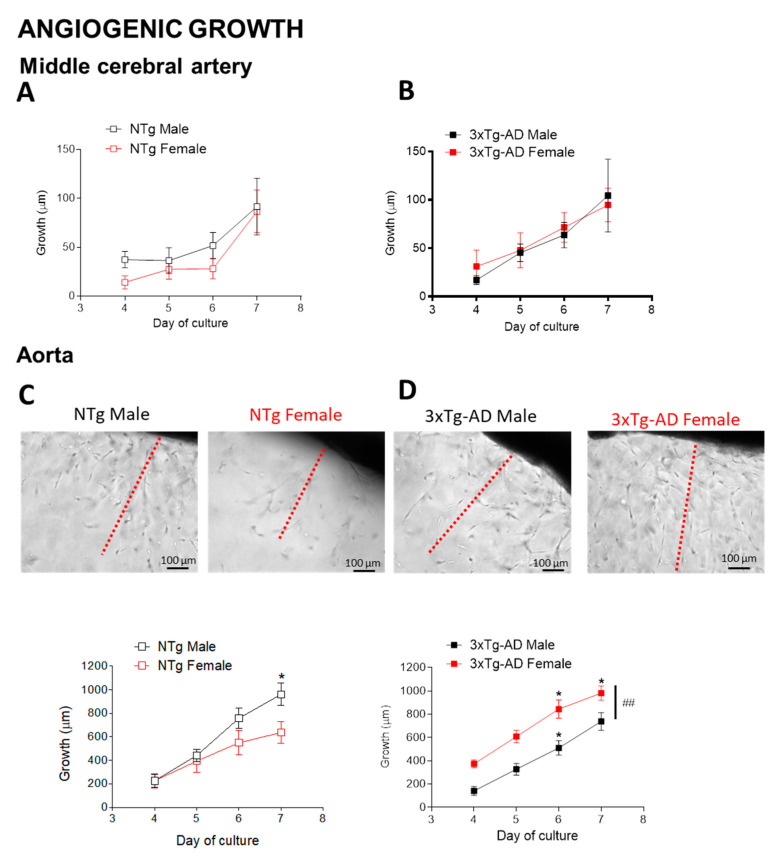
Influence of sex and genotype on the MCA and aortic angiogenic response. Analysis of neovessel growth progression in MCA from NTg (**A**) and 3xTg-AD (**B**) male and female mice. Representative images of neovessel sprouting (above) at day 7 (left) or 6 (right) and analysis of neovessel growth (below) in aorta from NTg (**C**) and 3xTg-AD (**D**) male and female mice. Red dotted lines represent the length of the longest vessel sprouting from the artery’s outer surface (starting point). Results are the mean ± SEM from NTg, male *n* = 6–7, female *n* = 4–5; 3xTg-AD, male *n* = 10, female *n* = 5. Data were analyzed by two-way repeated measures ANOVA with Tukey post-test. * *p* < 0.05, ## *p* < 0.01.

The middle cerebral artery (MCA) (Figure 7A,B) and the aorta (Figure 7C,D) was longitudinally measured at day 4, 5, 6, 7 after seeding in growth medium containing Matrigel. Sex did not modify MCA angiogenic growth either in NTg or 3xTg-AD mice, though female 3xTg-AD mice showed an enhanced (*p* < 0.01) growth from day 4 to 6 compared to female NTg. In the aorta, although NTg females showed an initially lower angiogenic growth than males, growth was significantly increased (*p* < 0.05) in 3xTg-AD females compared to 3xTg-AD males (Figure 7D) and NTg females (Figure 7C vs. 7D).

### 3.7. Arterial Properties

**Figure 8:**
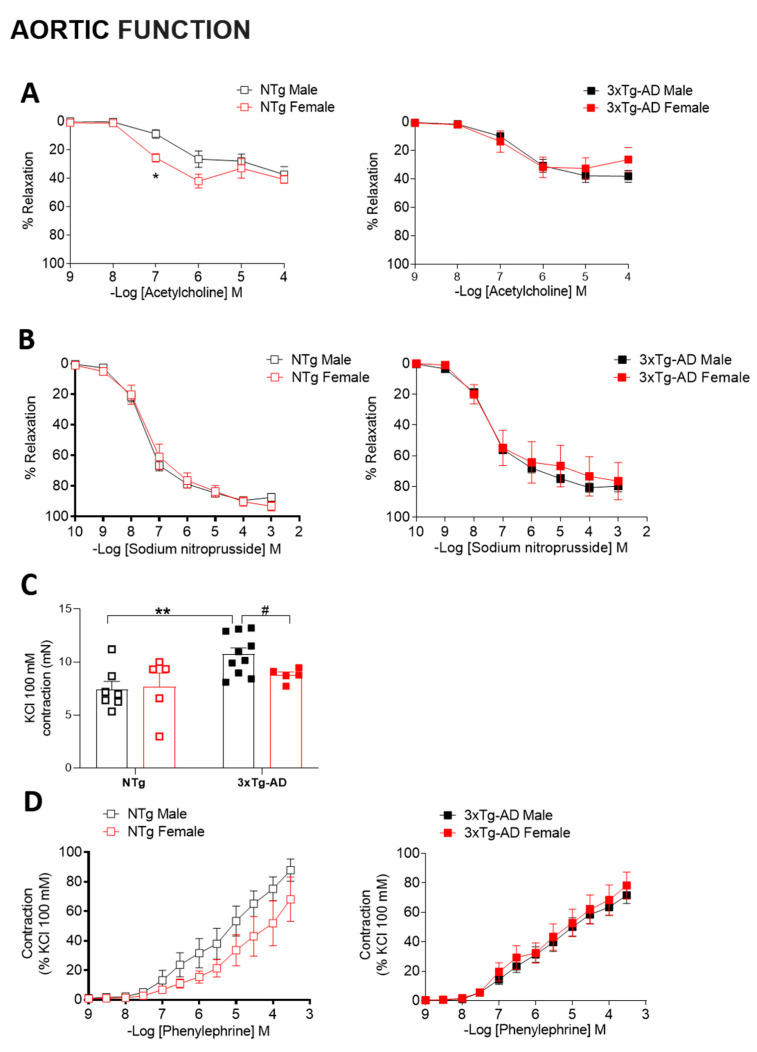
Influence of sex and genotype on aortic function. Concentration-dependent relaxations to acetylcholine (**A**) and sodium nitroprusside (**B**) in thoracic aortas from NTg and 3xTg-AD male and female mice. Contractions to KCL 100 mM (**C**) and concentration-dependent contractions to phenylephrine (**D**). Results are the mean ± SEM from NTg, male *n* = 7, female *n* = 5; 3xTg-AD, male *n* = 10, female *n* = 5. Data were analyzed by ×-way repeated measures (**A**,**B**,**D**) or regular (**C**) two-way ANOVA with Tukey post-test. * *p* < 0.05, ** *p* < 0.01, # *p* < 0.05.

Endothelium-dependent ACh-induced vasodilatation in the aorta of female NTg mice was slightly higher than NTg males, whereas no sex-dependent differences were found in 3xTg-AD mice (Figure 8A). Sodium nitroprusside relaxations were not affected by sex, either in NTg or 3xTg-AD mice (Figure 8B). However, these relaxations were impaired (*p* < 0.05) in 3xTg-AD compared to NTg males. These results suggest an impairment of smooth muscle relaxing responses in 3xTg-AD males. We subsequently measured contractile responses to KCl (100  mM) (Figure 8C) and Phe (Figure 8D). Responses to KCl were significantly higher (*p* < 0.01) in the 3xTg-AD genotype in males but not females, an effect that culminated in greater (*p* < 0.05) contractions in 3xTg-AD males than females (Figure 8C). Nevertheless, concentration-dependent contractions to Phe were not affected either by sex or genotype (Figure 8D).

### 3.8. Mental Health and Cardiovascular Function Correlates

**Figure 9:**
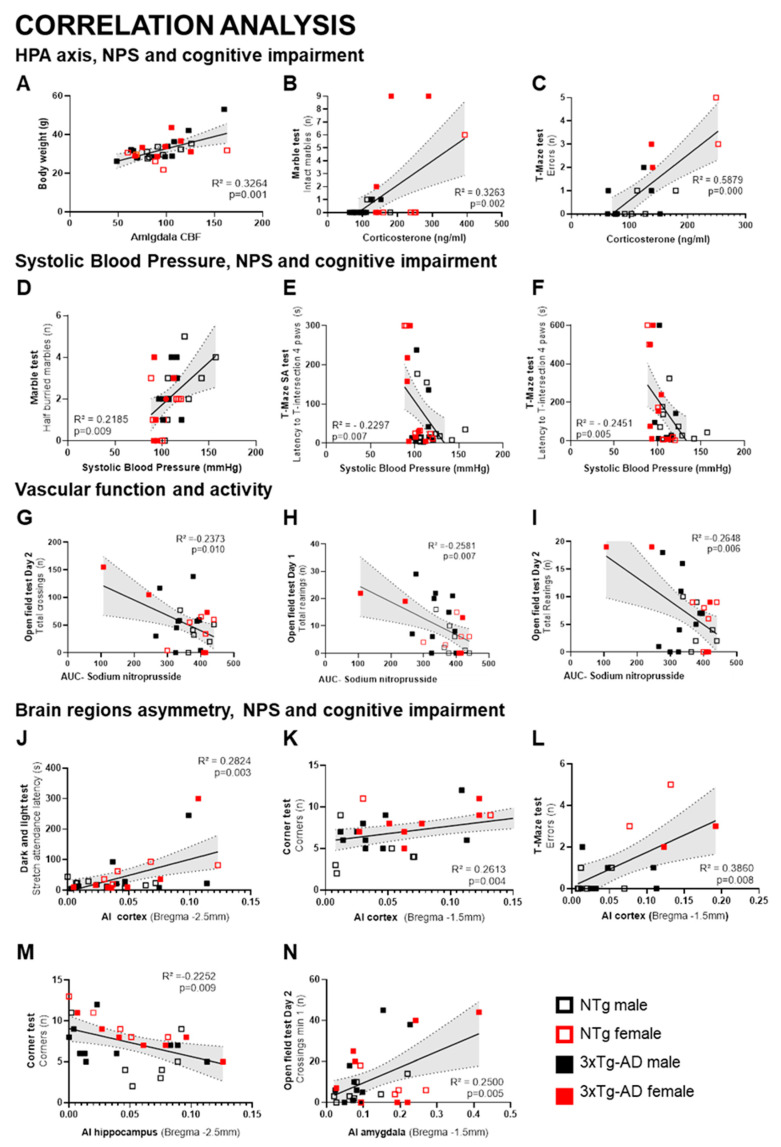
Mental health—cardiovascular function correlation analysis. Meaningful significant Pearson r correlations between behavior and cardiovascular function and HPA axis. (**A**) Amygdala CBF with body weight (**B**) corticosterone levels with the number of intact marbles, and (**C**) errors in the T-maze. (**D**) Systolic blood pressure with the number of half-buried marbles and (**E**) with the latency to arrive to T-intersection with 4 paws in TMSA test and (**F**) TM test. (**G**) Vascular function, as measured by the area under the curve (AUC) of sodium nitroprusside and the total horizontal activity in the first open-field test and (**H**,**I**) vertical activity in the two open fields. (**J**) Cortical asymmetry index (AI) and risk assessment behavior, (**K**) neophobia in the corner test, and (**L**) errors in the T-maze. (**M**) Hippocampal’s asymmetry index and neophobia in the corner test, (**N**) amygdala’s asymmetry index and neophobia in the second open-field test.

Behavioral correlates with cardiovascular function and HPA axis activation were analyzed. The most statistically significant correlations found when considering the whole sample of animals are illustrated in Figure 9. Amygdala CBF was positively correlated with body weight (Figure 9A, *p* = 0.001), and corticosterone levels with the number of intact marbles (Figure 9B, *p* = 0.002) and errors in the T-maze (Figure 9C, *p* < 0.001). Systolic blood pressure was positively correlated with the number of half-buried marbles (Figure 9D, *p* = 0.009) and negatively correlated with latency to arrive to T-intersection with four paws in the TMSA test (Figure 9E, *p* = 0.007) and TM test (Figure 9F, *p* = 0.005). Sodium nitroprusside relaxations (area under the curve), a measure of endothelium-independent relaxations, were negatively correlated with the total horizontal activity in the first open-field test (Figure 9G) and vertical activity in the two open-fields (Figure 9H, *p* = 0.007, and 9I, *p* = 0.006). Asymmetry of several brain regions was also correlated with behavior. Cortical areas were positively correlated with risk assessment behavior (Figure 9J, *p* = 0.003), neophobia in the corner test (Figure 9K, *p* = 0.004), and errors in the T-maze (Figure 9L, *p* = 0.008). Hippocampal’s asymmetry index (AI), defined as (right-left)/(right + left) * 100 was negatively correlated with neophobia in the corner test (Figure 9M, *p* = 0.009), while amygdala’s asymmetry index was positively correlated with neophobia in the second open-field test (Figure 9N, *p* = 0.005).

The functional correlates of the behavioral signatures were also analyzed for the corresponding group and are presented as supplementary data (supplementary material). Briefly, the increased and persistent neophobia in female 3xTg-AD mice, confirmed also in the open-field test, was correlated with Phe %KCL- pEC50 (r > −0.983, *p* < 0.003), area under the curve (AUC) (r = −0.983, *p* = 0.001), PEC50 (r = -0.983, *p* = 0.007), and KCL (r > −0.965, *p* < 0.008). The hyperactive pattern of male 3xTg-AD mice in the open-field test was correlated with the striatum (Bregma -1.5 mm) asymmetry index (AI) (r = 0.790, *p* = 0.006), systolic blood pressure (r = −0.910, *p* = 0.002), PEC50 Acetylcholine (% Phe) (r = −0.842, *p* = 0.002), and amygdala (Bregma −1.5 mm) AI (r> 0.776, *p* < 0.009). The disinhibitory behavior in male and female 3xTg-AD mice in the dark–light box was correlated with acetylcholine (% Phe)- area under curve (r= 0.707, +, *p* = 0.003); cortex (Bregma -2.5 mm) AI (r= 0.612, +, *p* = 0.009), acetylcholine (% Phe)- maximum effect (r = -0.679, +, *p* = 0.005); the frailty index FI (r= 0.616, +, *p* = 0.009) and global cerebral blood flow in the selected slices (r= 0.660, +, p = 0.004), striatum (r = 0.709, +, *p* = 0.001), caudate putamen (r = 0.671, +, *p* = 0.003), amygdala (r = 0.622, +, p = 0.009); striatum (Bregma −2.5 mm) AI (r = −0.766, *p* < 0.001). The slower speed of female 3xTg-AD mice in the water maze was correlated with the cerebral blood flow of whole brain (r = -0.878, *p* = 0.009), striatum (r = −0.893, +, *p* = 0.007), caudate putamen (r = -0.916, +, *p* = 0.004), and hippocampus (r = −0.906, *p* = 0.005). Finally, paradoxal performances in male 3xg-AD mice probably related to their emotional and physical comorbid conditions to cognitive function were found correlated with acetylcholine (% Phe)- PEC50 (r = 0.774, +, *p* = 0.009), sodium nitroprusside (% Phe)- PEC50 (r = 0.784, +, *p* = 0.007), cerebral blood flow of whole brain (r= 0.778, +, p = 0.008), striatum (r = 0.795, +, *p* = 0.006), caudate putamen (r = 0.807, +, *p* =0.005), and cortex (Bregma −1.5 mm) AI (r = 0.844, +, *p* = 0.002).

## 4. Discussion

The present work investigated the interaction between mental health and cardiovascular disease under a translational gender-medicine perspective. We used two strains of mice modeling normal (NTg) and neurodegenerative (3xTg-AD) aging, where first evidence of compromised small peripheral mesenteric resistance artery (MRA) properties was recently shown [21]. Worse physiologically relevant MRA structural and functional alterations of 3xTg-AD females suggested sex-dependent dysfunctions. We hypothesize that those findings would also extend to other cardiovascular health measures. Since the aging process is very heterogeneous [7], here, we studied two cohorts where females exhibited worse mortality rates than males since birth. We were interested in exploring brain–cardiovascular interaction mechanisms relevant for long-lived animals. Eight functional aspects were successively studied from middle age to natural death or euthanasia at 16 months. First, we determined the physical (including frailty) and behavioral (neuropsychiatric-like and cognitive) phenotypes of animals. Once the “mental health” of each animal was characterized, we determined their “cardiovascular phenotype” through systolic blood pressure, rCBF, angiogenesis, and arterial function. Survival was continuously monitored. Glucocorticoid levels, an indicator of HPA axis function, were analyzed from blood samples collected during the euthanasia. In the results, the analysis includes the sample size at each time point; so that the results of a certain functional analysis are those taking into account the highest sample available. The results agreed with those from the analysis performed only using the final sample size of survivors (censored data).

### 4.1. Sex- and Genotype-Dependent Mortality/Morbidity Paradox

People with AD show worse survival than the general old population, and deranged neuro-immuno-endocrine system in males could explain their worse survival than females despite their less bad neuropathological status [4,6]. At the translational level, we have consistently reported increased vulnerability of 3xTg-AD mice concurrent with impairment of the neuro-immuno-endocrine system and in agreement with this sex-dependent profile [11,12,38]. However, we found cohorts that offered a distinct survival scenario, with pairwise comparisons in the survival curves confirming an effect of sex factor with worse survival of females, independently of the genotype. 3xTg-AD mice females had a shorter lifespan than males, and sex differences were less pronounced in NTg mice. The retrospective analysis of survival indicated that both groups of females exhibited an early mortality window, starting as soon as 2–3 months of age. While the male sex has worse cardiovascular mortality rates than females, the burden of cardiovascular disease in the female sex is widely reported [39]. Less is known in this regard in Alzheimer’s disease. Therefore, the present cohorts offer an interesting experimental setting to study the morbidity/mortality paradox in surviving females. In this scenario, we were interested in studying the relationship between frailty, mental health, and cardiovascular phenotype.

Heterogeneity is found in the aging process, and prognostic tools to identify end-of-life dementia stages are difficult [7]. In NTg and 3xTg-AD mice, we described heterogeneity as part of the complexity of age-related scenarios [8,40,41]. The frailty index, a common tool to measure health status that seems to be sensitive to predict mortality [5], is a valuable tool in longevity and aging studies in mice [31]. In the general population, women usually present higher frailty scores and a reduced risk of mortality than men [42]. In contrast, higher frailty scores are recorded in men with AD [43]. In agreement, the Mouse Clinical Frailty Index, a translational adaptation of the frailty index data in humans [31], was increased in male 3xTg-AD mice compared to NTg counterparts, while females exhibited similar frailty scores. A worse sensorimotor function was also previously reported [8,10,12]. Using the same frailty index, other researchers also noticed that 3xTg-AD males show higher scores, with sex differences in health span predicting lifespan in the 3xTg-AD mouse model of AD [44].

### 4.2. Down-Regulation of HPA Axis Endocrine Status in Female 3xTg-AD Mice

At the endocrine level, mild hypercortisolemia is observed in AD patients [45], and the stimulation of the HPA can result in peripheral immune depression [46]. In the present work, the HPA axis exhibited sexual dimorphism, with higher levels of plasma corticosterone in females than males. The AD–genotype effect reduced plasmatic corticosterone levels in 3xTg-AD females, attenuating the sexual dimorphism in one degree of magnitude. These data agree with our first report in 15-month NTg and 3xTg-AD [12]. In all groups, the corticosterone levels were higher than those reported at 15 months, which could be due to the animals’ increased stress response to the experimental design, especially observed in females [47]. These corticosterone levels were similar to those observed in NTg and 3xTg-AD mice after chronic treatment with caffeine and vehicle [48].

### 4.3. Different Behavioral Signatures for Physical, Emotional, and Cognitive Phenotypes

The patterns of innate neophobia (fear of novelty) response shown by NTg mice were found broken in the 3xTg-AD genotype, and it is most prominently observable in the mutants with female sex, as consistently described since our first work [10] and confirmed afterwards [21,37,49]. We proposed increased neophobia, as delayed and reduced rearing, an early behavioral marker of the onset of behavioral and psychological symptoms of dementia (BPSD)-like symptoms since premorbid disease stages [28]. Here, the corner test was sensitive to old 3xTg-AD females’ genotype, where neophobia is enhanced. For the first time, we describe these patterns as also observable on the repeated test. 

In the open-field test, no sex differences were found in normal aging. In contrast, the frail male 3xTg-AD exhibited sustained activity, mostly as a thigmotaxis response and slower habituation pattern. A hyperactive pattern in frail 3xTg-AD males is also observed after social isolation [50]. The repetition of the test elicited reduced activity, in all the groups. In agreement with previous reports showing a 24 h long-term memory deficit in male 3xTg-AD mice at 2, 4, and 6 months of age [28], the behavioral response did not benefit from previous experience. Here, we show that these genotype effects in the immediate re-confrontation with the test are extensive to 14 months of age and mostly observed in females.

As shown by increased crossings and time in the lit area and grooming, disinhibitory behavior in the dark–light box confirmed a consistent BPSD-like phenotype in male and female 3xTg-AD mice. These disinhibitory patterns were first described at 4 months of age as part of the profile mimicking AD’s prodromal stage [51]. The marble test, assessing anxiety-like behaviors and screen drugs for obsessive-compulsive disorders and psychotic symptoms [52], also showed a specific pattern for females 3xTg-AD mice. 

In the paradigms for learning and memory in mazes [37], the number of animals failing to complete the T-maze indicates their aged status and/or poor motivation [8]. The latency to achieve the first goal of the test (crossing the intersection) has been related to immunosenescence and reduced survival [53]. This ceiling effect resulted in a sample of “successful animals” that equally performed the spontaneous alternation task. However, these animals committed errors attributed to working memory (revisiting explored areas) when assessed in a more complex task (the forced-choice paradigm) in a sex-specific manner. Here, females of both genotypes spent more time choosing the right choice and committed more errors. 

The water maze’s performance was strongly determined by genotype differences in the swimming speed (slower in 3xTg-AD mice) mostly found among females. Motor features can be discarded (the frail animals were males), but the swimming performance can reflect their emotional status in an aquatic environment known to be anxiogenic for mice [54]. To control this factor, the distance covered was used instead of the latency. Two learning and memory tasks differing on the level of complexity and involving short (15 min) and long-term (24 h) memory were used. The day-by-day and trial-by-trial analysis showed a notorious aged profile compared to previous reports in young [10,11,28,37,49,51,55] or old animals [8,38,48,56]. Worse performance of female NTg mice was observed on the visual perceptual learning task. Long-term spatial reference learning and memory deficits were shown by all the groups in their first day of place learning task where the hidden platform, located in a reversed location, had to be found. Finally, the two-day place task indicated paradoxical better performances in male 3xTg-AD mice, which could be explained by the strong need that a frail animal may have to find and remember a safe place.

### 4.4. Increased MRI-ASL Regional Cerebral Blood Flow in 3xTg-AD Survivor Females

In the present study, we evaluated CBF in five different brain regions, namely, hippocampus, cortex, striatum, caudate putamen, and amygdala using arterial spin labeling (ASL), a magnetic resonance imaging (MRI) technique for non-invasive measurements of cerebral blood blow. The results indicated sex- and brain-region-associated changes in CBF. Among all, 3xTg-AD female mice survivors had increased CBF in the cortex and hippocampus as compared with their wild-type counterparts. 

Although CBF alteration seems to be involved in AD pathogenesis, the perfusion patterns remain unclear, since both hypoperfusion and hyperperfusion have been described in different brain areas and involved in different brain functions [57]. 

Oxidative stress, inflammation, and cerebrovascular disease have been suggested to be involved in AD. In one of our recent collaborative studies in 3xTg-AD mice, we have reported that the number of β-amyloid (Aβ) plaques in the hippocampus and entorhinal cortex at advanced stages of the disease was higher in females than in males [58]. Interestingly, co-localization of hypoxic areas and Aβ plaques in the hippocampus and entorhinal cortex were observed only in females. In the present study, the increased CBF in the cortex and hippocampus in female survivors suggests a potential compensatory hemodynamic mechanism in end-of-life dementia, which is sex- and brain-region-dependent. This is interesting to note, since recent work in APP/PS1 transgenic mice at the early stages of AD has shown longitudinal changes in regional CBF, indicating age- and brain-region-dependent alterations of cerebral blood flow [59]. At the clinical level, an increased CBF has also been observed in patients at preclinical stages of AD when cognitive performance is still preserved, suggesting a compensatory response to the accumulation of Aβ pathology [60]. Moreover, the reduction in CBF seems to be an important factor contributing to the cognitive dysfunction associated with dementia. One study performed with APP/PS1 mice in the late stages of the disease reported that a treatment that consists of the increase in the cerebral blood flow improves cognition [61]. 

### 4.5. Sex- and Brain-Region-Dependent Asymmetry in the MRI-ASL Regional Cerebral Blood Flow

Brain structural and functional asymmetry in health/disease is an emerging field. The neurodegeneration of subcortical structures is not symmetric, with neuroimaging studies reporting volumetric regional, hemispheric asymmetries. Thus, asymmetric hippocampal atrophy has been recently reported in normal aging, mild cognitive impairment, and AD [62]. A whole-brain analysis revealed increased neuroanatomical asymmetries in dementia for the hippocampus and amygdala and is proposed as a powerful imaging biomarker [63]. At the translational level, hippocampal asymmetry was important in rodents for acquiring spatial reference memory, retaining working memory [64], and some features of non-spatial learning [65]. At the neurochemical and molecular levels, left–right hippocampal asymmetry has been demonstrated for the glutamatergic system [65]. We have just provided the first evidence of brain atrophy asymmetry in male 3xTg-AD mice, thus modeling that found in human patients with AD [50]. However, little is known about the alterations in CBF hemisphere asymmetries. In the present work, the MRI-ASL rCBF results unveiled, for the first time, the asymmetry between left–right hemispheres in the female’s cortex, in the hippocampus of control males, and 3xTg-AD females, as well as in the striatum of control females. Therefore, the present results show asymmetry between left–right hemispheres in the 3xTg-AD model and aging mice, in both sexes (but mostly in females) and in cortical and subcortical structures. Moreover, to ensure that these detected asymmetries in rCBF measurements were not affected by differences in mice’s head positioning (rotation respect to the sagittal plane) or differences in signal-to-noise ratio (SNR) or contrast-to-noise ratio (CNR) between hemispheres, a SNR/CNR quality check analysis was performed that showed no statistically significant differences among the experimental groups (data not shown). This modeling will be useful for the translational development and assessment of the preventive/ therapeutic interventions and those of the risk factors and hazards and monitoring disease progression.

### 4.6. Improved Vascular Profile in 3xTg-AD Survivor Females

Hypertension is a risk factor to develop cognitive impairment and dementia [66] and is associated with the pathological manifestations of Alzheimer’s disease [67]. Although it is more prevalent in men, a recent study demonstrated that midlife hypertension increases the risk of dementia in women but not in males [68]. Arterial pressure increases in a sex- and age-specific manner similar to humans, showing sex differences until 14 months [69]. In agreement with this, the sex effect was observed in this study, with males presenting an increased systolic blood pressure. Although our results did not demonstrate a higher systolic blood pressure in 3xTg-AD mice than NTg mice, as it has been observed in AβPP/PS1 mice [70], a correlation between higher levels of systolic blood pressure and decreased regional cerebral blood flow was observed in 3xTg-AD mice.

AD is associated with cerebral and peripheral artery dysfunction, a process that can lead to altered blood flow to the brain, which in turn can increase the risk of developing AD or impair AD pathology [71,72]. In mice models of AD disease, both peripheral large [18,19,20] and small [21] artery dysfunction have been reported. A previous study showed decreased ACh- and sodium-nitroprusside-dependent relaxations in conjunction with increased endothelin-1 contractions in aortas from 11-month-old male 3xTg-AD mice [18]. Consistently, in the 16-month-old male 3xTg-AD compared to NTg mice of the present study, similar low levels of ACh-induced relaxations, impairments of sodium nitroprusside responses, and enhanced contractions to KCl 100 mM were observed. Notably, 3xTg-AD female mice survivors had an improved vascular profile.

On the one hand, although 3xTg-AD females showed similar low levels of ACh relaxation than males, relaxations to sodium nitroprusside and contractions to KCl were unaltered compared to NTg mice. On the other hand, the angiogenic growth capacity of the MCA and aorta was higher in 3xTg-AD compared to NTg females, and 3xTg-AD females showed greater aortic angiogenesis than 3xTg-AD males. In a precedent work, the anxious-like behavioral profile correlated with vascular alterations in small mesenteric arteries from 15-month-old 3xTg-AD female mice [21]. Altogether, we suggest that the vascular profile is tightly linked to the overall health status, especially in female 3xTg-AD mice.

The increased angiogenic response correlated with increased CBF in the cortex and hippocampus in 3xTg-AD compared to NTg females, an effect that is consistent with the concept that angiogenesis, would be a compensatory response to impaired CBF in AD [22]. These findings agree with previous studies that found increased angiogenesis in the hippocampus, midfrontal cortex, substantia nigra, pars compacta, and locus ceruleus of post mortem human AD brains [73]. It is worth noting that increased angiogenesis, which has been associated with the presence of Aβ [24,74,75,76], could be linked to increased vascular permeability, especially under pro-oxidant and pro-inflammatory environments, such as in AD [23,24]. A non-invasive method was used to measure blood pressure due to the old age of mice. However, a high-fidelity blood pressure phenotyping method (e.g., radiotelemetry) would be necessary to confirm blood pressure data. Besides, we used the thoracic aorta (i.e., a large conductance artery) as a model for generic peripheral artery function. Therefore, the present study is limited by the lack of information on vascular function in peripheral resistance arteries, which might reflect better what happens at the level of cerebral vasculature.

### 4.7. Behavioral Correlates Mental Health and Cardiovascular Measurements

Correlation analysis through all the components suggests functional interactions between NPS and cognitive impairment with amygdala and HPA axis. The vascular function correlated with activity, while the left and right hemispheres’ asymmetry of rCBF with NPS and cognitive impairments. Increased activation of the amygdala in 3xTg-AD mice [55] could explain the specific correlation between amygdala CBF and body weight. Corticosterone level correlations agree with our first report on the increase in glucocorticoid levels concomitantly to increased anxiety and peripheral immune dysfunction [12] and recent work of other laboratories [77]. Systolic blood pressure correlated positively with the number of half-buried marbles and negatively correlated with latency to cross the T-intersection and TM test, which are indicators of a worse neuro-immunoendocrine function, accelerated aging, and premature death in mice [53]. These correlates agree with mice chronically subjected to high blood pressure being more active in the open-field and faster in a spontaneous alternation test [78]. Recently, we demonstrated the correlation between peripheral small vessel properties and anxiety in both NTg and 3xTg-AD mice, mostly in females [21]. Here, we show that the animals with lower endothelial-independent vasodilatations to sodium nitroprusside (i.e., lower muscle relaxation capacity) were the most active in the open-field test, in both non-goal- (horizontal activity) and goal-directed (rearing) behaviors [79,80]. The correlation was consistent, as also observed in the repeated test**.** Regarding relative cerebral blood flow and neuropsychiatric and cognitive impairment, the cortex was the better area correlated with behavior. Cortex hemisphere asymmetry correlated with risk assessment (stretch attendance), neophobia in the corner test, and worse memory performance in the T-maze test. Correlation between asymmetry and neophobia was also observed in the hippocampus and amygdala. Since testing the pairwise correlations between dozens of variables without multiple comparison correction may involve a high risk of type-1 error, only those which are meaningful, obtained a maximum statistical significance (p < 0.001), and could be verified with close-related variables were considered. Despite our aim to highlight and explore the sex-based differences, the statistical power of the sample size per sex did not allow to compare whether the correlations between these variables were different between male and female mice and thus modulated by the sex. Overall, this correlation analysis allows for finding meaningful functional correlations between behavioral responses related to the levels of anxiety, cognition and locomotor activity, and cardiovascular measurements. Especially, the present work provides pieces of evidence of the brain regions’ asymmetry of both males and females with normal and AD-neurodegenerative aging correlation with neuropsychiatric symptoms and cognitive deficits.

### 4.8. Future Research Directions

The present study suggests a potential compensatory hemodynamic mechanism in end-of-life dementia, which is sex-dependent. Those vascular adaptations observed in 3xTg-AD female mice survivors might provide clues to understand potential vascular targets for pharmacological and non-pharmacological interventions.

## Figures and Tables

**Table 1:** Synopsys of main genotype and sex effects in the physical condition and behavioral phenotype.

Domains	Tests	Effect	Between Groups Differences	Figure
Physical Condition				
Survival	Survival curve	Sex **	3xTg-AD females vs. males	Figure 1A
Frailty	Frailty index	Genotype ***	3xTg-AD males vs. NTg males	Figure 1D
Weight	Body weight	Genotype *	3xTg-AD females vs. NTg females	Figure 1B
HPA axis	Corticosterone	Genotype*; Sex**	Females vs. males	Figure 1C
**Behavioral Phenotype**				
**Neuropsychiatric-Like Domain**				
Neophobia	CT,OF	Sex *		Figure 2A,B
Hyperactivity	OF	Genotype *	3xTg-AD males vs. NTg males	Figure 2B
Disinhibition	DLB	Genotype *		Figure 2C
**Cognitive Domain**				
Long-term memory	OF2	Genotype *		Figure 2B
Working memory	TM	Sex ***	Females vs. males	Figure 3B
Swimming speed	MWM	Genotype *	3xTg-AD females vs. NTg females	Figure 3C
Paradoxical performance	MWM	Genotype *	3xTg-AD males vs. NTg males	Figure 3C

Domains studied, tests used (CT, corner test; OF, open-field test; OF2, open-field test day 2; DLB, dark–light box test and MWM, Morris water maze). Statistics: genotype and sex effect; between groups differences, * *p* < 0.05; ** *p* < 0.01; *** *p* < 0.001 and related figures.

## Data Availability

Not applicable.

## Supplementary Materials

Supplementary materials can be found at https://www.mdpi.com/2227-9059/9/2/111/s1. Supplementary data: Mental health and cardiovascular function correlates.

## Abbreviations

3xTg-AD	Triple transgenic mice
AD	Alzheimer’s disease
AI	Asymmetry index
ASL	Arterial spin labeling
AUC	Area under the curve
BLA	Basolateral amygdala
BPSD	Behavioral and psychological symptoms of dementia
CBF	Cerebral blood flow
CPu	Caudate putamen
CNR	Contrast-to-noise ratio
CT1	Corner test—Day 1
CT2	Corner test—Day 2
DALYs	Disability-adjusted life years
DLB	Dark–light box test
G	Genotype
HC	Hippocampus
HALE	Healthy life expectancy
HPA	Hypothalamic–pituitary–adrenal
MB	Marble test
MCA	Middle cerebral artery
MCFI	Mouse Clinical Frailty Index
MRA	Mesenteric resistance arteries
MRI	Magnetic resonance imaging
MWM	Morris water maze
NPS	Neuropsychiatric symptoms
NTg	Non transgenic mice
OF1	Open-field test—Day 1
OF1n	Open-field test—Day 1, minute n of the test
OF2	Open-field test—Day 2
OF2n	Open-field test—Day 2, minute n of the test
PT n n	Place task—Day, trial n of the test
RMA	Repeated measures ANOVA
ROI	Region of interest
S	Sex
SNR	Signal-to-noise ratio
T	Time
TM	T-maze
TMSA	T-maze spontaneous alternation
