# Supplementary material for: Sex-Dependent End-of-Life Mental and Vascular Scenarios for Compensatory Mechanisms in Mice with Normal and AD-Neurodegenerative Aging
Source: Biomedicines. 2021 Jan 24;9(2):111. doi: 10.3390/biomedicines9020111 (PMC7911097; doi:10.3390/biomedicines9020111)
Supplement: Supplementary file 1 [file biomedicines-09-00111-s001.pdf]

Mental health and cardiovascular function correlates

The correlations between the different variables studied were evaluated with Pearson's correlation. The *p*<0.01 are illustrated.

**Physical phenotype**	**rCBF**	**Arterial properties**
**Systolic blood pressure**	**Angiogenesis**	

(+) Positive correlation (-) Negative correlation

CORNER TEST- Day 1

- Total corners: Asymmetry index (AI) hippocampus, AI Cortex (Bregma -1.5mm); AI Caudate Putamen

OPEN FIELD TEST- Day 1

- Latency of movement : Whole brain CBF; all areas CBF
- Exit of the center latency: Whole brain CBF; Cortex CBF
- Latency to enter the periphery: AUC Acetylcholine (% Phe)
- 1st minute rearings: AUC Sodium nitroprusside (% Phe); pEC50 Sodium nitroprusside (% Phe); maximum effect Sodium nitroprusside (% Phe)
- 2nd minute rearings: AUC Sodium nitroprusside (% Phe)
- Total rearings: AUC Sodium nitroprusside (% Phe)

CORNER TEST- Day 2

- Total corners: AI amygdala

OPEN FIELD TEST- Day 2

- 1st minute crossings: AUC Sodium nitroprusside (% Phe), maximum effect Sodium nitroprusside (% Phe); AI amygdala
- 2nd minute crossings: AI amygdala
- 4th minute crossings: AUC Acetylcholine (% Phe), pEC50 AUC Acetylcholine (% Phe)
- Total crossings: AUC Sodium nitroprusside (% Phe)
- 1st minute rearings: AUC Sodium nitroprusside (% Phe); maximum effect Sodium nitroprusside (% Phe)
- 2nd minute rearings: AI amygdala
- Total rearings: AUC Sodium nitroprusside (% Phe)
- Number of groomings: Frailty index

DARK AND LIGHT TEST

- Latency of movement: Cortex CBF
- Stretch attendance latency: AUC Acetylcholine (% Phe), maximum effect Acetylcholine (% Phe), AI Cortex (Bregma -2.5mm)
- Time in Light Body weight

- Time of groomings: Whole brain CBF, AI striatum (Bregma -2.5mm)

MARBLE TEST

- Intact Marbles: Corticosterone
- Half buried Marbles: Systolic blood pressure

T- MAZE TEST SPONTANEOUS ALTERNATION TASK

- T- intersection latency: Systolic Blood Pressure, AI Cortex
- T- intersection with 4 paws latency: Systolic Blood Pressure

T MAZE

- T-intersection with 4 paws latency: Systolic Blood Pressure
- Error number: Corticosterone; pEC50 Phenylephrine (% KCl), AI Cortex (Bregma -1.5mm)

MORRIS WATER MAZE

- CUE 2 speed: Cortex CBF
- CUE 4 Time: maximum effect Phenylephrine (% KCl); amygdala CBF
- CUE 4 speed: Striatum CBF
- Mean CUE speed Whole brain CBF
- Place Task 1.1 speed: Whole brain CBF, Striatum CBF, Hippocampus CBF
- Place Task 1.3 speed: Whole brain CBF, all areas CBF
- Mean Place Task 1 speed: pEC50 Phenylephrine (% KCl), Whole brain CBF, Striatum CBF
- Place Task 2.2 Distance: Frailty index, AI Caudate Putamen
- Place Task 2.3 Distance: Corticosterone
- Place Task 2.4 Distance: angiogenic growth aorta
- Place Task 2.2 Speed: pEC50 Phenylephrine (% KCl)
- Place Task 2.4 Speed: pEC50 Phenylephrine (% KCl), Whole brain CBF, Striatum CBF
- Mean place task 2 distance KCl, angiogenic growth aorta
- Mean Place Task 2 speed pEC50 Phenylephrine (% KCl)

Physical phenotype

Body weight: Amygdala CBF

The functional correlates of the behavioral signatures were also analyzed for the corresponding group.

Neophobia 3xTg-AD female Corner test (CT)/Open Field test (OF)

- Corner test_ Day 1_ Total rearings: pEC50 phenylephrine %KCl
- Corner test_ Day 2_ Total corners: Area under the curve phenylephrine (AUC) %KCl/ PEC50
- Open Field test_ Day 1_ 1st minute crossings: KCl
- Open Field test_ Day 1_ 4th minute crossings: KCl
- Open Field test_ Day 1_ 1st minute rearings: KCl
- Open Field test_ Day 1_ 2nd and 4th minute rearings: Phenylephrine (% KCl)
- Open Field test_ Day 2_ 4th minute crossings: KCl
- Open Field test_ Day 2_ 1st minute rearings: AUC Phenylephrine (% KCl)
- Open Field test_ Day 2_ 3rd and 4th minute rearings pEC50 Phenylephrine (% KCl)

Hyperactive pattern of male 3xTg-AD mice in the Open-field test

- Open Field test_ Day 1_ 1st minute crossings: Asymmetry Index (AI) striatum
- Open Field test_ Day 2_ 1st minute crossings: **systolic blood pressure**
- Open Field test_ Day 2_ 2nd minute crossings: PEC50 Acetylcholine (% Phe)
- Open Field test_ Day 2_ 1st, 2nd minute and total crossings AI amygdala

Disinhibitory behavior in male and female 3xTg-AD mice in the Dark-light box test

- Stretch attendance latency: AUC Acetylcholine (% Phe); AI cortex (Bregma -2.5mm)
- Stretch attendance number: maximum effect Acetylcholine (% Phe)
- Time in light: Frailty index
- Time doing grooming: Whole brain CBF, Striatum CFB, Caudade Putamen CBF, Amygdala CBF; AI striatum (Bregma -2.5mm)

Slower speed of female 3xTg-AD mice in the Morris water-maze

- Place task 1.3 speed: Whole brain CBF, Striatum CBF, Caudate Putamen CBF
- Place task 2.3 speed: Hippocampus CBF
- Place task 2.4 speed: Striatum CBF

Paradoxical performances of male 3xg-AD in Morris water-maze

- CUE 2 Time: PEC50 Acetylcholine (% Phe)
- CUE 3 Time: Whole brain CBF, Striatum CBF, Caudate Putamen CBF
- Place Task 1.3 Time/Distance: AI cortex (Bregma -1.5mm)
- Place Task 2.1 Distance: PEC 50 Sodium nitroprusside (% Phe)
